# Intention to leave profession, psychosocial environment and self-rated health among registered nurses from large hospitals in Brazil: a cross-sectional study

**DOI:** 10.1186/s12913-016-1949-6

**Published:** 2017-01-10

**Authors:** Daiana Rangel de Oliveira, Rosane Härter Griep, Luciana Fernandes Portela, Lucia Rotenberg

**Affiliations:** 1 Laboratory of Health and Environment Education, Oswaldo Cruz Institute (IOC/FIOCRUZ), Pav Lauro Travassos. Avenida Brasil, 4365, Manguinhos, Rio de Janeiro, 21040-360 Brazil; 2 National School of Public Health, Oswaldo Cruz Foundation (ENSP/FIOCRUZ), Pav Lauro Travassos, Avenida Brasil, 4365, Manguinhos, Rio de Janeiro, 21040-360 Brazil

**Keywords:** Nursing shortage, Intention to quit nursing, Workers’ health, Hospital

## Abstract

**Background:**

Nurses’ intention to leave their profession is a worldwide concern. Studies have shown that it can take the form of a chain reaction: many nurses first leave the unit, then the hospital, and finally the profession. Organisation and other labour factors, personal and conjunctural, have been associated with the intention to quit nursing. This study aimed to examine the factors associated with the intention to leave the profession among registered nurses (RNs) at large public hospitals in Brazil.

**Methods:**

This was a cross-sectional study, conducted from 2010 to 2011: all RNs at Rio de Janeiro’s 18 largest public hospitals (>150 beds) were invited to participate. The study sample comprised 3,229 RNs (82.7% of those eligible), who answered a self-completed, multidimensional paper questionnaire. The outcome was defined as thoughts of leaving the profession sometimes a month or more. We based the analyses on hierarchical logistic regression models, considering three blocks of determinants: socio-demographic data (block I), occupational factors (block II), and health conditions (block III).

**Results:**

Of the study population, 22.1% indicated the intention to leave the profession. In the final model after adjustment, the variables associated with the intention to leave were as follows: male sex (odds ratio [OR] = 1.65), not holding a leadership position (OR = 1.28), highly demanding work (OR = 2.49), passive work (OR = 2.10), effort-reward imbalance (OR = 2.00), poor self-rated health (OR = 1.92), over-commitment to the job (OR = 1.87), and poor supervisor support (OR = 1.33). The likelihood of expressing the intention to leave increased with age (OR = 0.98 for the oldest).

**Conclusions:**

Self-rated health and factors connected with the work environment, particularly those that generate psychosocial strain, were most strongly associated with the intention to leave the profession. From the profiles of nurses who wished to leave the profession, we found that for many people who go into nursing—especially men and younger entrants—their prospects of remaining in the profession are poor. The potential role of psychosocial job characteristics and self-rated health indicates the need for long-term action involving all stakeholders, i.e. managers, employers, and workers.

## Background

Nurses constitute the largest workforce in health-care services [1, 2]. That force will become increasingly necessary, particularly because of accelerating populating ageing and the mounting burden of chronic non-communicable diseases [1, 3]. Internationally, this situation poses a series of challenges to health services with respect to human resource management in nursing. The lack of investment in [1] and growing indifference to the profession [4] are but two of the critical issues related to the scarcity of nurses. In addition, psychosocial strain [5–7] and high turnover [3, 8] are strongly associated with intentions to leave the profession [9–11].

Many studies have identified the intention to quit nursing as a predictor of the final decision to leave the profession [10, 12]. The Nurses’ Early Exit Study (NEXT) [13] found that the final decision to leave the profession is generally taken within 6 months of actually doing so; it also determined that about 80% of those who left the profession had begun to consider the move seriously within the previous 12 months.

In the past 10 years, the scarcity and high turnover [14] of nurses have been acknowledged as a problem worldwide and researched most intensely in Europe [10, 13, 15, 16]. Following economic crises, most European countries have reduced their budget provisions for health-care professionals, thereby narrowing the scope for engaging new personnel. At the same time, it has been predicted that the numbers of health-care professionals will decline drastically over the coming decade owing to workforce ageing [1]. One explanation for this trend, according to Buerhaus et al. [4], is that opportunities for women are expanding outside nursing, leading to a decline in the number of young women entering the profession. This diminishing inclination in the younger generations to choose nursing as a career has resulted in a continuously ageing workforce [4, 15, 17].

The intention to leave nursing has often been investigated, particularly using quantitative methods, to identify associations with a number of variables [18]. Prominent among the socio-demographic factors that displayed an association with the intention to leave nursing were younger age, male sex, and higher level of schooling [5, 9, 13, 19].

In addition, occupational factors associated with the intention to leave the profession include working at a hospital, poor support from supervisors, lack of meaning in the work, conflicting roles, and absence of development opportunities [5, 13]. Nurses’ intention to leave the profession has been shown to associate with psychosocial job strain evaluated on the demand-control-support (DCS) [20] and effort-reward imbalance (ERI) [21] models. In this regard, the intention to quit is a phenomenon connected with work organization and occupational environment issues. Some studies have shown that the intention to leave can occur in the form of a chain reaction, with many nurses leaving first their unit, then their hospital, and ultimately the profession [22, 23]. In that context, physical burdens [5], long working hours [5], the number of patients per nurse [24], and psychosocial strain [10, 25] have all shown associations with the intention to leave.

With respect to health factors, the NEXT results indicated that burnout syndrome was significantly associated with the intention to leave the profession, a finding that agrees with those of Goodin [15] and Hayes et al. [10]. An appropriate state of health and good self-rated health are known to be essential to performing well at work. In that respect, it is unsurprising that poor self-rated health is associated with intention to leave the profession [13, 26]. It should be noted that health problems can be caused by poor working conditions [27], which can prompt intentions to leave.

Studies about the intention of nurses to leave the profession have been mainly restricted to European countries; an exception here is Engeda et al. [28]. This has hindered policy-making efforts to retain nurses in their profession. Brazil is a country where labour factors exert a notable influence on nurses leaving their profession. According to Health at a Glance [2] figures, there were only 1.5 nurses/1,000 people in Brazil in 2011; the mean for Organisation for Economic Co-operation and Development countries was 8.8 nurses/1,000 people.

In Brazilian hospitals, nurses are required to manage care: they plan nursing actions and schedules and they also provide materials. In addition, they provide care to more severe patients—all of which demands specific expertise and decision making [29]. Further, nurses’ duties include preventive health education, health planning, and administrative and management activities. However, proper performance of these duties, which are intended to assure quality care, is jeopardised in Brazil’s major hospitals by factors related to organisation and working conditions [30]. In this regard, studies have shown that nurses working in hospitals are continually subjected to job stressors, such as staffing shortages, which entail a build-up of duties and overwork. Additional factors include shiftwork, conflicting and ambiguous roles, little participation in decision making, lack of a career or wage plan, a feeling of injustice in labour relations, and conflicts with colleagues or institutions [31, 32].

The present study deals with registered nurses working at the 18 largest public hospitals in Rio de Janeiro, Brazil. The conceptual framework we adopted to address the intention to leave the profession was based on that proposed by Hasselhorn et al. [13]; those authors used their approach to analyse the reasons, circumstances, and consequences related to nurses in 10 European countries leaving the profession [13]. Our model accommodates the fact that leaving the profession is the result of a complex process, in which several factors can act together in different directions. Push factors correspond to aspects that adversely affect workers: they favor the desire to stop working (e.g., work conflicts, poor health). Pull factors are attractive incentives that can lead to early departure from a profession, such as regulated early retirement. Our conceptual model encompasses three broad dimensions: (1) demands, including aspects related to both the work environment and private life, that affect workers cumulatively or suddenly; (2) individual resources to deal with demands, e.g. health and age; and (3) final outcome, i.e. intent to stay or leave (exit a particular job or exit nursing). We supposed that the alternatives for the workers (i.e. socio-economic and organizational aspects related to opportunities for education, regulation for early retirement, or better pay) would influence the outcome. We address those three dimensions in the present study, which aims to examine the factors associated with the intention to leave the profession among registered nurses at large public hospitals in Brazil.

## Methods

### Design and participants

We conducted a cross-sectional study at the 18 largest public hospitals (>150 beds) in the Rio de Janeiro municipal area. Eligible workers were all registered nurses currently working at the selected hospitals. Each nurse was contacted personally by a team of interviewers (in most cases, nurses themselves), who explained the objectives of the study and invited them to participate. We collected data from April 2010 to December 2011.

### Data collection

We based data collection on a multidimensional, self-administered paper questionnaire filled in by the workers at their workplace; it included questions relating to the nursing work environment, health-related behaviour, and socio-demographic data. We refined the questionnaire in five rounds of pre-tests.

### Variables definition

Our analysis addressed the three broad dimensions described in the Background [13]: (1) the demands of work and home environments; (2) individual resources as exposure variables; and (3) the intention to leave or exit the profession as the outcome.

### Exposure variables

The socio-demographic variables were as follows: sex; age (continuous); self-reported skin colour (categorised into “black”, “white”, and “other”, the latter comprising indigenous background, Asian background, and other mixed background); marital status (“never married or never cohabited”, “married or cohabiting”, and “divorced or widowed”); education level (“graduate”, “postgraduate/specialization course”, or “postgraduate/doctorate or master’s degree”); monthly per capita income (“up to US$125.00”, “$126.00–$208.00”, or “$209.00–$667.00”); and domestic overload, which considered the number of people in the household (excluding the subject) and the degree of responsibility for housework (cleaning, cooking, washing, and ironing) [30].

Occupational variables were as follows: years working in nursing (continuous); work schedule (day or night worker); weekly work hours (continuous); number of jobs; type of contract (permanent or temporary); having worked as a health-care professional before becoming a nurse; and holding a leadership position. Psychosocial job factors were assessed using two different approaches. First, we used the ERI model [21], which addresses the lack of reciprocity between effort expended and reward received in the workplace. This model also assesses the over-commitment dimension, which is based on the assumption that a high level of personal commitment increases the risk of reduced health. Second, we used the DCS model [20], according to which job strain occurs when people are psychologically overloaded when they are deprived of control over their work environment, as assessed by the quadrant term (low strain, passive job, active job, or high strain). This scale also considers the social support from supervisors and from co-workers. The response categories for both instruments were a Likert scale ranging from 1 (strongly disagree) to 4 (strongly agree), and they were subsequently reverse-coded afterwards if necessary. The following were the Cronbach’s alpha coefficients for the ERI dimensions: effort (0.71); reward (0.60); and over-commitment (0.81). The Cronbach’s alpha coefficients for DCS dimensions were as follows: psychological demands (0.71); emotional demands (0.59); job control (0.75); supervisor support (0.74); and co-worker support (0.81).

The health-related variables were as follows: self-rated health (evaluated by the question, “Generally, compared with people your age, what do you consider your state of health?”) and disease-related absenteeism (measured by the question, “In the past 12 months, how many whole days have you been off work because of health problems, medical appointments, or tests?”).

### Outcome variable

The intention to leave nursing was evaluated by asking, “How often during the past year have you thought about giving up nursing?” The answer categories were as follows: never; sometimes a year; sometimes a month; sometimes a week; and every day. For the purposes of analysis, we categorised the sample into two levels [13]: often (respondents considering leaving the sometimes a month, sometimes a week, or every day); and not often (those who never thought of leaving the profession or considered leaving sometimes a year).

### Data analysis

We based our analyses on a hierarchical model, which described the relationships between the exposure variables and outcome. First, we performed bivariate analyses to test the association between independent variables and intention to leave nursing. We selected variables that showed a statistical significance with *P* ≤ 0.20 for the multiple logistic regression analysis. We hierarchised the selected independent variables into three blocks of determinants: (1) block I (socio-demographic factors: sex, age, marital status); (2) block II (occupational factors: holding a leadership position, having worked as a health-care professional before becoming a nurse, type of contract, weekly working hours, ERI, over-commitment, work schedule, quadrants of the DCS model, supervisor support, and support from colleagues); and (3) block III (self-rated health: sickness absenteeism).

Second, we conducted multivariate logistic regression according to a hierarchised approach to variable entry for odds ratio (OR) and 95% confidence interval (CI) adjustment [31]. In the regression model, we included block I variables, which we adjusted for each other: we retained those that showed *p* ≤ 0.05 in the model—even if they lost statistical significance as variables in the other blocks. We adopted the same strategy for the variables in blocks II and III. It should be noted that we did not include the variable “years working in nursing” in the logistic regression model because it was strongly correlated with age.

In accordance with the above-mentioned theoretical model [13], we defined the reference category as that with the lowest expected risk for intention to leave the profession. We performed statistical analyses using the Statistical Package for the Social Sciences (IBM SPSS, version 19).

### Ethics, consent, and permissions

The studied protocol was approved by the ethics committee of the Oswaldo Cruz Foundation (472/08) and by the ethics committees of the hospitals where the research was conducted. The purpose of the study was briefly explained to participants, who were informed that involvement was voluntary and that they could withdraw at any time with no negative implications. All the information obtained was confidential, and there was no possibility of identifying the participants in the database. The participants signed informed consent forms.

## Results

Of 3,904 eligible subjects, 3,229 joined the study (82.7% of the total number of registered nurses [RNs]). The 675 (17.3%) subjects who did not participate were as a result of refusal (*n* = 478), being unavailable for multiple visits over a period of 2 months (*n* = 128), and absence through vacation (*n* = 69).

The study population displayed the following characteristics: 87.3% female; mean age, 40 years (standard deviation [SD] ± 10.0 years); 55% declared themselves “white”; and 57.5% reported being in a stable personal relationship. The mean duration of working in nursing was 15 years (SD ± 9.7 years); two-thirds of participants regularly worked nights; and the mean working week was 55 h (SD ± 21.0 h). Among the participants, 22.1% declared the intention to leave the profession: this variable was significantly associated with younger age (*p* < 0,001) and being single (*p* < 0,001). There was also a borderline association with being male (Table 1).

**Table 1:** Bivariate association between socio-demographic characteristics and intention to leave nursing

Characteristics	Often^a^				
	n	%	OR	95% CI	*p*
Gender					
Female	609	21.6	1.00		0.057
Male	106	25.8	1.26	(0.99–1.60)	
Self-reported skin color					
White	386	21.7	1.00		0.970
Black	76	21.9	1.01	(0.76–1.33)	
Other	231	22.1	1.02	(0.85–1.23)	
Age (mean ± SD)	37.4 (±9.5)		0.96	(0.96–0.97)	<0.001
Domestic overload (mean ± SD)	13.9 (±15.3)		0.57	(12.8–15.1)	0.378
Marital status					
Married/cohabiting	415	22.6	1.00		<0.001
Never married/cohabiting	194	25.4	1.16	(0.96–1.41)	
Divorced/widowed	97	16.4	0.67	(0.52–0.85)	
Education level					
University degree	181	22.8	1.00		0.567
Postgraduate/specialization course	481	22.2	0.97	(0.80–1.17)	
Postgraduate/doctorate or master’s degree	45	19.5	0.82	(0.57–1.18)	
Monthly *per capita* income (US$)					
Up to 125.00	193	21.6	1.00		0.308
From 126.00 to 208.00	210	21	0.96	(0.77–1.20)	
From 209.00 to 667.00	226	23.8	1.13	(0.90–1.40)	

^a^vs. not often

Crude analyses showed a higher likelihood for the intention to leave nursing among the following participants: not working in health care before becoming nurses; not holding a leadership position; having spent less time in the profession; working more hours per week; working nights; working under contract; being exposed to a strong ERI; reporting over-commitment to work; having a highly demanding job; and receiving poor support from supervisors and colleagues (Table 2). Among participants who reported poor self-rated health, the likelihood of intending to leave was three times higher than among those reporting good health (Table 3).

**Table 2:** Bivariate association between work-related factors and intention to leave nursing

Characteristics	Often^a^				
	n	%	OR	95% CI	*p*
Worked as a healthcare professional before becoming a nurse					
Yes	144	18.4	1.00		0.004
No	562	23.3	1.35	(1.10–1.65)	
Holds a leadership position					
No	513	23.8	1.00		0.003
Yes	189	19.1	1.32	(1.09–1.59)	
Years working in nursing (mean ± SD)	13.3	(±9.5)	0.97	(0.96–0.98)	<0.001
Weekly hours worked (mean ± SD)	57.5	(±22.5)	1.00	(1.00–1.01)	0.031
Work schedule					
Day worker	246	19.7	1.00		0.007
Night worker	469	23.7	1.27	(1.06–1.51)	
Number of jobs					
One	228	21.4	1.00		0.759
Two	393	22.4	1.06	(0.88–1.28)	
Three or more	94	22.8	1.08	(0.83–1.43)	
Type of contract					
Permanent	434	20.4	1.00		<0.001
Temporary	757	25.6	1.34	(1.12–1.59)	
Effort–reward imbalance					
Low imbalance	293	43.4	1.00		<0.001
High imbalance	382	56.6	3.55	(2.97–4.24)	
Overcommitment					
No	370	16.7	1.00		<0.001
Yes	326	34.9	2.68	(2.25–3.19)	
Demand-Control Model					
Low strain	76	9.1	1.00		< 0.001
Active job	162	21.5	2.73	(2.03–3.65)	
Passive job	134	21.4	2.72	(2.01–3.69)	
High strain	294	37.5	5.98	(4.54–7.89)	
Supervisor support					
High	273	16.4	1.00		<0.001
Low	1057	71.4	2.04	(1.72–2.43)	
Coworker support					
High	490	20	1.00		<0.001
Low	210	29.1	1.65	(1.36–1.98)	

^a^vs. not often

**Table 3:** Bivariate association of self–reported health and absenteeism with intention to leave nursing

Characteristics	Often^a^				
	n	%	OR crude	95% CI	*p*
Self-rated health					
Good/very good	387	18.3	1.00		<0.001
Regular	224	25.7	1.54	(1.27–1.86)	
Poor/very poor	92	40.4	3.01	(2.26–4.02)	
Absenteeism in the past 12 months					
None	358	20.8	1.00		0.116
Up to 9 days	200	22.6	1.11	(0.91–1.35)	
10 days or more	147	24.8	1.25	(1.00–1.56)	

^a^vs. not often

In the final model, after adjustment for all variables, male nurses were more likely (OR, 1.65; 95% CI, 1.24–2.19) to think often of leaving nursing. Age was a protective factor among all the assessed blocks: the intention to leave was less likely in older than in younger participants.

With respect to labour characteristics, participants who had not worked in health care before becoming nurses were 57% (OR, 1.57; 95% CI, 1.23–2.00) more likely to consider leaving the profession. Subjects who did not hold a leadership position were 28% more likely (OR, 1.28; 95% CI, 1.03–1.59) to do so.

The intention to leave was twice as likely in the group with a strong ERI as in the group with a weak imbalance (OR, 2.00; 95% CI, 1.60–2.48). In addition, participants reporting excessive commitment were 87% more likely to consider leaving the profession (OR, 1.87; 95% CI, 1.51–2.32). Nurses in jobs classified as highly demanding were 149% more likely to intend leaving than the low-demand group (OR, 2.49; 95% CI, 1.80–3.44). We also observed higher likelihoods in the passive and active job groups: the intention to leave was, respectively, 110 and 54% greater than in the low-demand group.

With regard to the association between support from supervisors and the intention to leave, we observed a 33% higher likelihood among those who reported poor support (OR, 1.33; 95% CI, 1.09–1.64). Workers with poor self-rated health were 92% more likely to often consider leaving nursing than those with good self-rated health (OR, 1.92; 95% CI, 1.38–2.67; Table 4).

**Table 4:** Logistic regression analysis of factors associated with intention to leave nursing

Characteristics	OR crude (IC95%)	Block I OR (IC95%)	Block II OR (IC95%)	Block III OR (IC95%)
Gender				
Female	1.0	1.0	1.0	1.0
Male	1.26 (0.99–1.60)	1.40 (1.08–1.83)	1.63 (1.24–2.17)	1.65 (1.24–2.19)
Age	0.96 (0.96–0.97)	0.97 (0.96–0.98)	0.98 (0.97–0.99)	0.98 (0.97–0.99)
Worked as a healthcare professional before becoming a nurse				
Yes	1.0		1.0	1.0
No	1.35 (1.10–1.65)		1.55 (1.22–1.97)	1.57 (1.23–2.00)
Holds a leadership position				
No	1.0		1.0	1.0
Yes	1.32 (1.09–1.59)		1.28 (1.03–1.59)	1.28 (1.03–1.59)
Effort-reward imbalance				
Low imbalance	1.0		1.0	1.0
High imbalance	3.55 (2.97–4.24)		2.05 (1.65–2.54)	2.00 (1.60–2.48)
Overcommitment				
No	1.0		1.0	1.0
Yes	2.68 (2.25–3.19)		1.97 (1.60–2.43)	1.87 (1.51–2.32)
Demand–Control Model				
Low strain	1.0		1.0	1.0
Active job	2.73 (2.03–3.65)		1.57 (1.14–2.17)	1.54 (1.11–2.13)
Passive job	2.72 (2.09–3.69)		2.14 (1.53–2.98)	2.10 (1.50–2.93)
High strain	5.98 (4.54–7.89)		2.60 (1.89–3.60)	2.49 (1.80–3.44)
Supervisor support				
High	1.0		1.0	1.0
Low	2.04 (1.72–2.43)		1.35 (1.10–1.66)	1.33 (1.09–1.64)
Self-rated health				
Good/very good	1.0			1.0
Regular	1.54 (1.27–1.86)			1.18 (0.94–1.46)
Poor/very poor	3.01 (2.26–4.02)			1.92 (1.38–2.67)

## Discussion

The proportion of workers who reported often intending to leave nursing (22.1%) was higher than that observed in the countries included in the NEXT [13]: there, the proportions ranged from 8.8% (Netherlands) to 20.7% (Italy). Compared with the results of the present study, another study in European countries showed modest percentages of dissatisfaction with nursing work. With regard to the global scarcity of nurses, which reflects dissatisfaction within the profession and the consequent leaving rate, Buchan & Aiken [32] found the shortage to be related to precarious working conditions. They suggested that proper workforce planning and allocation, better career support, and effective retention policies could address this problem. In this regard, as a result of work overload, the economic situation in developing countries is likely to make the intention to leave the profession more common than in developed countries. For example, mainly owing to low wages, Brazilian nurses usually have a second job. Accordingly, they have very long weekly working hours (56 h/week and 61 h/week for women and men, respectively) as shown by Fernandes et al. [33] in a previous investigation on the same sample of RNs analysed in the present study.

In this study, men were more dissatisfied with the profession than women. Gender relations in nursing have been gaining increasing attention in the specialist literature—both because historically the profession is associated with women [34] and because of men’s increasing participation in the profession [35]. Borkowski et al. [36] observed greater job dissatisfaction among the men in their sample: the authors found that in their job, male nurses encountered more resistance from those in leadership positions, female colleagues, and patients. Evans & Frank [34] observed that male nurses live in constant defence of their professional choice, their value to nursing, and their sexuality. Conversely, as evidenced by Williams [37], the choice of an essentially female profession may be motivated by expectations of rapid promotion to administrative positions rather than working directly with patients. Accordingly, it is fair to conjecture that our results may have been influenced by perceptions of unmet professional expectations contributing as a source of dissatisfaction among male nurses.

Our finding about the greater likelihood of younger nurses reporting the intention to leave the profession supports the results of Aiken et al. [38] and Wieck et al. [39]. Price & Mueller [9] noted that younger nurses are more exposed to repetitive tasks, participating less in decision making, lacking knowledge of their work, being paid less, and having fewer close friends in the workplace. These factors, they argued, could contribute to greater dissatisfaction with the profession among the youngest age groups. Older nurses may be considered to have survived the most critical periods of their careers [5] and thus express less intention to leave.

With regard to having worked previously in the health-care field, the practical knowledge and mastery of techniques thus gained may possibly facilitate adaptation to nursing activities; this would contribute to a lower likelihood of intending to leave the profession. For this group, becoming a nurse in Brazil may be seen to offer professional advancement and better earnings [40], which may also tend to contribute to reducing the likelihood of wanting to leave the profession.

Our finding that the intention to leave the profession was more frequent among participants not occupying leadership positions is in line with the results of Cortese [11]. After examining the hospital work environment and intention to leave nursing, Cortese observed that nurses who did not hold the position of coordinator were more likely to consider leaving both the hospital and the profession. Cortese argued that his findings were influenced by organisational aspects of the job, such as job status, wages, and interpersonal relations. It should be noted that in the present sample, nurses occupying leadership positions were not required to remain in that function. As a result, it can be inferred that if the participants regarded the job as wearing or stressful, they could relinquish the leadership position without necessarily leaving nursing.

Psychosocial job strain appeared to be a strong predictor for the observed results. Specifically, with regard to the ERI model, Jian et al. [7] determined that in addition to poor prospects of wage increases, little chance of promotion, and a lack of recognition, low job reward may be a decisive factor in the intention to leave the profession. The greater likelihood of wanting to leave the profession among nurses who report over-commitment to the job may be related to the greater effort in performing their duties; this would exacerbate frustrations with received rewards and make them more likely to leave the profession [7, 41].

The results from the quadrants of the DCS model corroborate the findings of Hayes et al. [10, 25], who identified psychosocial job strain as an important predictor of nurses wishing to quit. The authors did not find that high psychological demand alone affected the intention to leave. However, they determined that exposure to high psychological demand combined with low job control (which is characteristic of highly demanding jobs) did increase the likelihood of nurses wanting to leave the profession. Supervisor support, one of the dimensions evaluated using the DCS model, also displayed an association with the intention to leave nursing. Indeed, evidence from the literature shows that the relationship with coordinators can both prompt nurses to leave [42, 43] and encourage them to stay in the profession [44]. However, Estryn-Behar [45] argued that changes in leadership style alone are insufficient to increase the retention of nurses in the profession. For greater effectiveness, support from supervisors has to be accompanied by significant changes in specific work conditions that influence the intention to leave.

In this study, self-rated health was strongly associated with the intention to leave; it is considered a single-item measure of health status and a consistent indicator for morbidity and mortality [46, 47]. Another important datum, which corroborates the findings of the present analysis, is the association between poor self-rated health and early retirement among nurses [27]. Although the outcome analysed here was the intention to leave nursing, current health status can be assumed to play a strong part in the decision to quit. In this regard, we believe that additional longitudinal studies will be more effective in investigating the relation between the intention to leave the nursing profession and workers’ health and work environment factors.

### Limitations of the study

Our findings derived from a cross-sectional study, and so data on exposure and outcome were assessed simultaneously; therefore, no time relation can be established among the events studied. This characteristic of a cross-sectional study design does not permit causality measurements. In addition, the possibility of reverse causality cannot be discarded. For example, the association between psychosocial job strain and intention to leave the profession may not have resulted from the influence of the psychosocial environment on job satisfaction, but on an inverse relationship: those nurses who indicated a stronger intention to leave the profession may have been more sensitive to the psychosocial environment. It should be noted that our findings cannot be generalised to the overall population of nurses working in the hospital system since our study did not include private hospitals. Further, we did not apply a sampling procedure that would furnish data on a representative sample of nurses working at public hospitals.

## Conclusions

From the profiles of nurses who wished to leave the profession, we found that for many people who go into nursing—especially men and younger entrants—their prospects of remaining in the profession are poor. Among the variables investigated, those relating to psychosocial job characteristics and self-rated health displayed the strongest associations with the intention to leave. Obviously, these factors call for long-term action involving all stakeholders, i.e. managers, employers, and workers.

Given the worldwide importance of retaining nurses in the profession and with a view to identifying actions to avert early exit from the profession, this study contributes to the discussion of the factors associated with the wish to leave nursing. The intention to leave is a useful indicator, which can be assessed continuously at the organisation level so as to inform human resource planning in nursing. That evaluation can reveal trends among different types of health-care institutions: when conducted in detail, it will allow this phenomenon to be prevented more effectively.

## Abbreviations

CI: Confidence interval
DCS: Demand-control-social support
ERI: Effort-reward imbalance
NEXT: Nurses’ early exit study
OR: Odds ratio
RN: Registered nurse
SD: Standard deviation
